# Supplementary material for: Linking biochemical and cellular efficacy of MERS coronavirus main protease inhibitors
Source: bioRxiv. 2026 Feb 21:2026.02.20.707097. Preprint. [Version 1] doi: 10.64898/2026.02.20.707097 (PMC12934682; doi:10.64898/2026.02.20.707097)
Supplement: Supplementary file 1 [file media-1.pdf]

Supplementary Information for "Linking biochemical and cellular efficacy of MERS coronavirus main protease inhibitors"

Van N. T. La,[†] Noa Lahav,[‡] Mario Rodriguez,[¶] Randy Diaz-Tapia,[¶] Briana McGovern,[¶] Jared Benjamin,[¶] Haim Barr,[‡] Kris M. White,[¶] Lulu Kang,[§] John D. Chodera,[‖] and David D. L. Minh[*,⊥]

†*Department of Biology, Illinois Institute of Technology, Chicago, IL 60616, USA*

‡*The Weizmann Institute of Science, Rehovot, 7610001, Israel*

¶*Icahn School of Medicine at Mount Sinai, Department of Microbiology and Global Health and Emerging Pathogens Institute, New York, NY 10029, USA*

§*Department of Mathematics and Statistics, University of Massachusetts Amherst, Amherst, MA, 01003, USA*

‖*Computational and Systems Biology Program, Memorial Sloan Kettering Cancer Center, New York, NY, USA*

⊥*Department of Chemistry, Illinois Institute of Technology, Chicago, IL 60616, USA*

E-mail: dminh@illinoistech.edu

Contents

Figure S1. Convergence of percentiles of the Bayesian posterior for fitting ES datasets **5**

Figure S2. 1D marginal distributions for ES fitting of MERS-CoV MPro **6**

Figure S3. 2D joint marginal distributions for pairs of binding free energies of fititng ES dataset of MERS-CoV MPro **7**

Figure S4. Convergence of percentiles of the Bayesian posterior of key parameters for fitting ES and ASAP-0000214 datasets **8**

Figure S5. Fit of the model to ES and ESI4c datasets for ASAP-0000214 **10**

Figure S6. 1D marginal distributions for binding free energies of fitting ES and ASAP-0000214 dataset of MERS-CoV MPro **11**

Figure S7. 2D joint marginal distributions for pairs of binding free energies of fititng ES and ASAP-0000214 dataset of MERS-CoV MPro **12**

Figure S8. Heat map of the correlation matrix estimated from the Bayesian posterior for free energies of fitting ES and ASAP-0000214 datasets of MERS-CoV MPro **13**

Figure S9. Linear correlation between $\Delta GK_{I,D}$, $\Delta G_{S,DI}$, and $\Delta G_{I,DI}$ of fitting ES and ASAP-0000214 datasets MERS-CoV MPro. **13**

Figure S10. 1D marginal distributions for rate constants of fitting ES and ASAP-0000214 datasets of MERS-CoV MPro **14**

Figure S11. 2D marginal distributions for pairs of rate constants of fitting ES and ASAP-0000214 datasets of MERS-CoV MPro **14**

Figure S12. **1D marginal distributions for enzyme concentrations of fitting ES and ASAP-0000214 datasets of MERS-CoV MPro** **14**

Figure S13. **2D joint marginal distributions for pairs of some binding free energies of fitting ES and all ESI4c datasets** **16**

Figure S14. **Heat map of the correlation matrix estimated from the Bayesian posterior for some binding free energies of fitting ES and all ESI4c datasets** **16**

Figure S15. **Fit of the model to ES and all ESI4c datasets** **16**

Figure S16. **Convergence of percentiles of the Bayesian posterior for shared parameters for fitting ES, all ESI4c, and 3 ESI1c datasets** **18**

Figure S17. **2D joint marginal distributions for pairs of some binding free energies of fitting ES, all ESI4c, and 3 ESI1c datasets** **19**

Figure S18. **Heat map of the correlation matrix estimated from the Bayesian posterior for some binding free energies for fitting ES, all ESI4c, and 3 ESI1c datasets** **19**

Figure S19. **Ratios of rate constants of MERS-CoV MPro** **19**

Figure S20. **Convergence of percentiles of the Bayesian posterior for key parameters for fitting one ESI1c dataset** **21**

Figure S21. **Fit of the model to ESI1c datasets** **22**

Figure S22. **Correlogram of inhibition, control, dimer pIC90 and cellular pEC90** **28**

Table S1. **Correlation matrix of biochemical pIC90 and cellular pEC90 by Pearson R, Spearman ρ, and Kendall τ** **29**

Table S2. Correlation matrix of biochemical pIC90 and cellular pEC90 by RMSD and aRMSD **29**

Table S3. p-value in comparison of correlation for $pIC50$s **30**

Table S4. p-value in comparison of correlation for $pIC90$s **31**

Fitting of ES datasets

Figure S1. Convergence of percentiles of the Bayesian posterior for fitting ES datasets

10,000 samples were drawn from the Bayesian posterior using the NUTS sampler. Lines correspond to the 5-th (blue circle), 25-th (green square), 50-th (red diamond), 75-th (cyan upward triangle) and 95-th (magenta downward triangle) percentile. The error bars, which are too small to be visible, are standard deviations estimated by 100 bootstrapping samples.

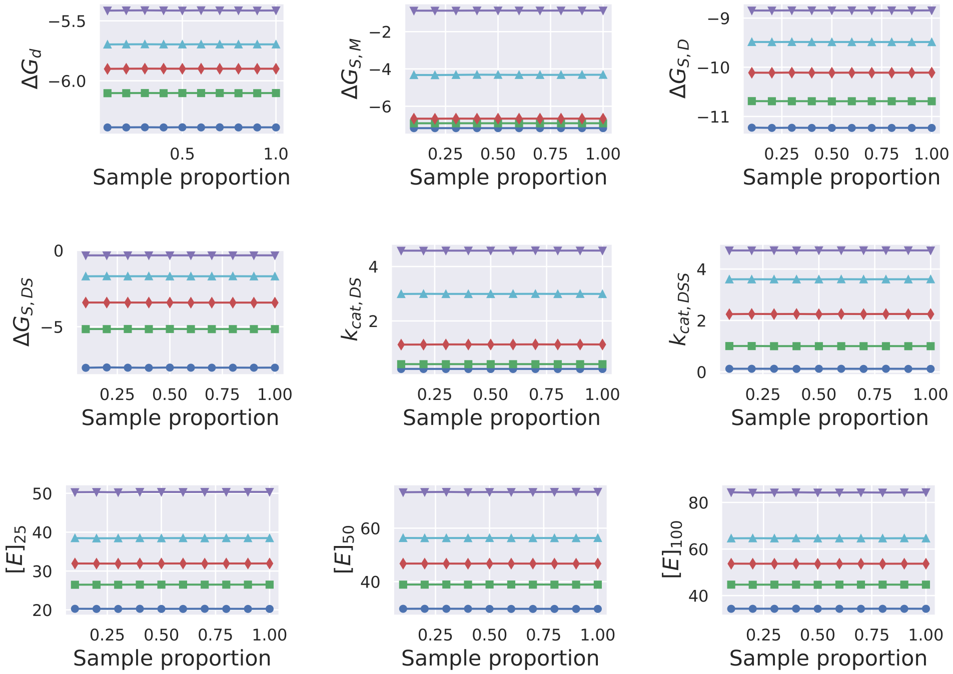

Figure S2. Representative 1D marginal distributions for ES fitting of MERS-CoV MPro

1D marginal probability densities for parameters were estimated based on 10,000 MCMC samples generated from the Bayesian posterior for all datasets. Red bars represent 95% HDIs. The green triangle marks the median of the posterior.

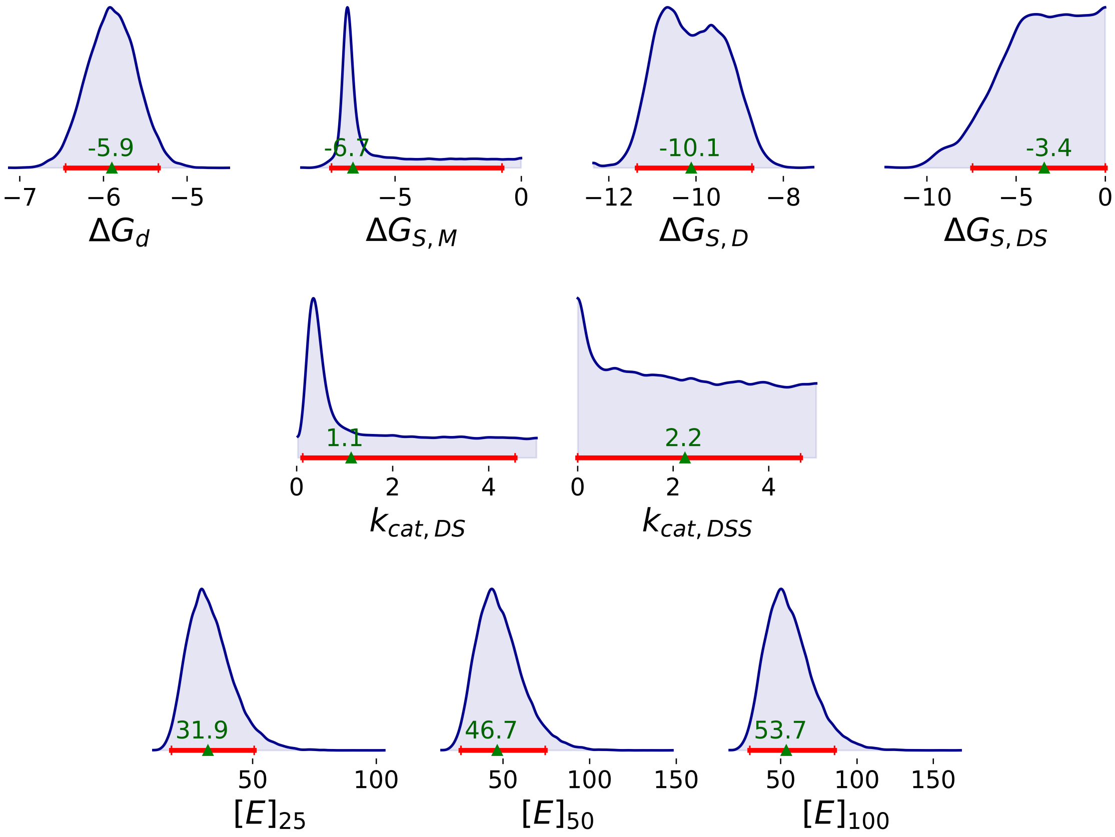

Figure S3. 2D joint marginal distributions for pairs of binding free energies of fitting ES datasets of MERS-CoV MPro

2D joint marginal probability densities were estimated based on 10,000 MCMC samples generated from the Bayesian posterior for all datasets.

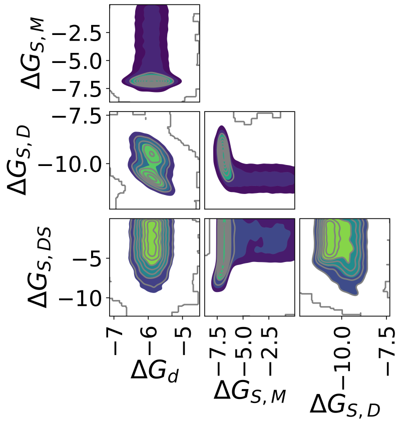

Fitting of ES + one ESI4c datasets

Figure S4. Convergence of percentiles of the Bayesian posterior of key parameters for fitting ES and ASAP-0000214 datasets

10,000 samples were drawn from the Bayesian posterior using the NUTS sampler. Lines correspond to the 5-th (blue circle), 25-th (green square), 50-th (red diamond), 75-th (cyan upward triangle) and 95-th (magenta downward triangle) percentile. The error bars, which are too small to be visible, are standard deviations estimated by 100 bootstrapping samples.

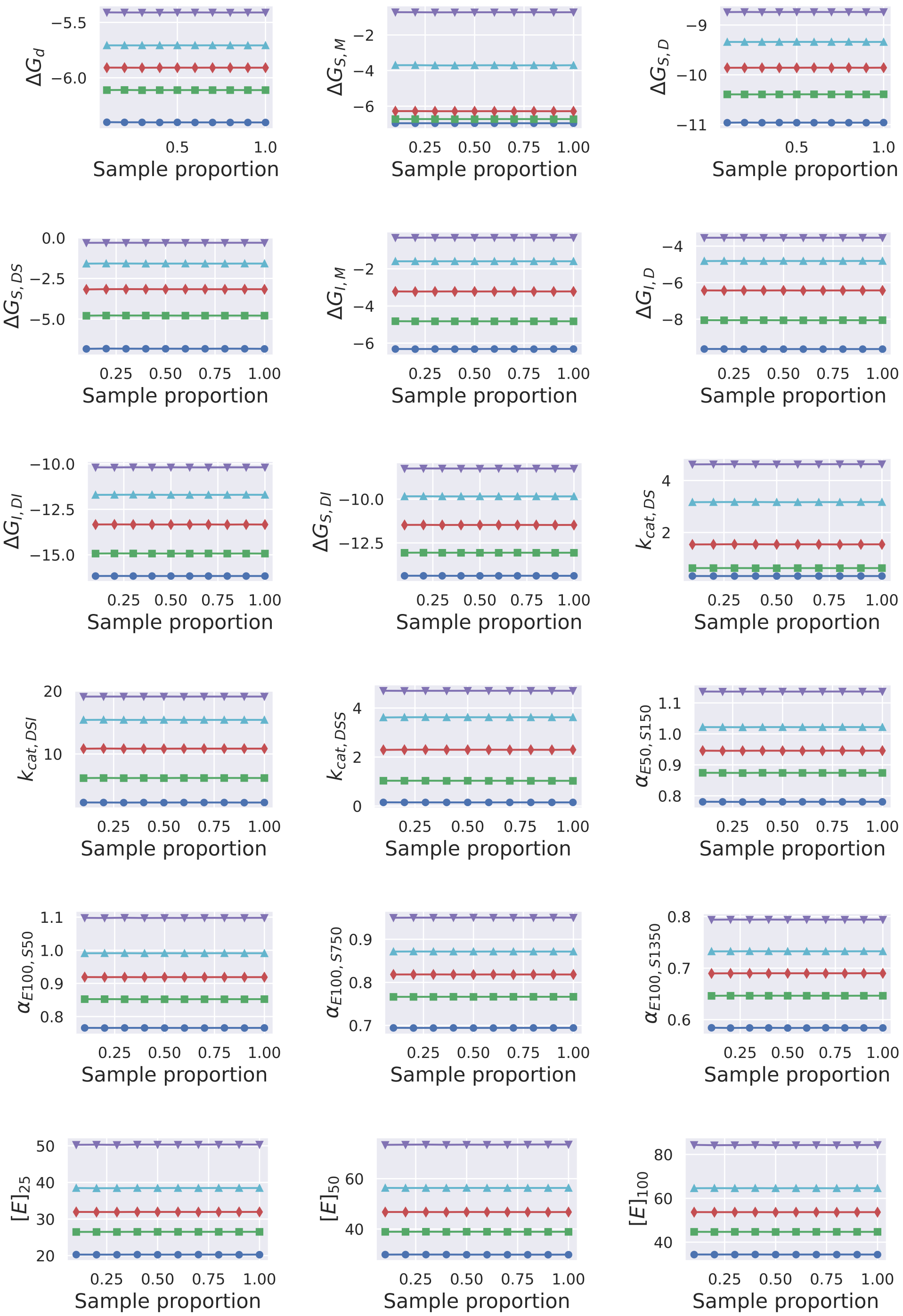
ΔG_d
$\Delta G_{S,M}$
$\Delta G_{S,D}$
$\Delta G_{S,DS}$
$\Delta G_{I,M}$
$\Delta G_{I,D}$
$\Delta G_{I,DI}$
$\Delta G_{S,DI}$
$k_{cat,DS}$
$k_{cat,DSI}$
$k_{cat,DSS}$
$\alpha_{E50,S150}$
$\alpha_{E100,S50}$
$\alpha_{E100,S750}$
$\alpha_{E100,S1350}$
$[E]_{25}$
$[E]_{50}$
$[E]_{100}$
Sample proportion

Figure S5. Fit of the model to ES and ESI4c datasets for ASAP-0000214

Dots are the observed response. X axes are concentrations (M). The theoretical response $y_n*(\boldsymbol{\theta}^{MAP})$ is represented by the dashed line, where $\boldsymbol{\theta}^{MAP}$ is the MAP estimate, the mean of the posterior prediction is solid line, and the 95% posterior predictive interval is the shaded region. Figure a: 100 nM (brown), 50 nM (red), and 25 nM (black) of the enzyme. Figure b: enzyme 100 nM, substrate 1350 nM (blue); enzyme 100 nM, substrate 750 nM (orange); enzyme 50 nM, substrate 150 nM (purple); enzyme 100 nM, substrate 50 nM (green); enzyme 50 nM, substrate 550 nM.

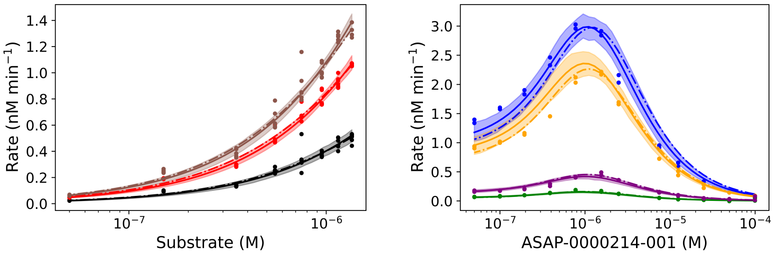

(a) (b)

Figure S6. 1D marginal distributions for binding free energies of fitting ES and ASAP-0000214 datasets of MERS-CoV MPro.

1D marginal probability densities for binding free energies were estimated based on 10,000 MCMC samples generated from the Bayesian posterior for all datasets. Red lines represent 95% HDIs. The green triangle marks the median of the posterior.

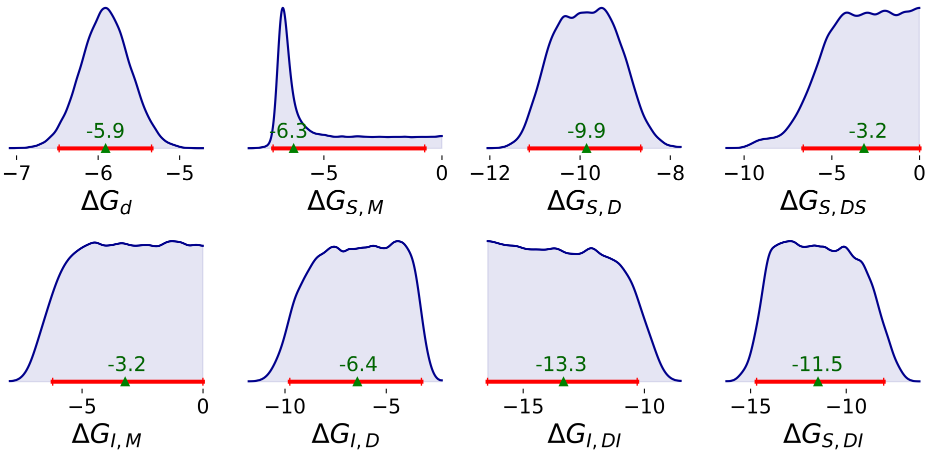

Figure S7. 2D joint marginal distributions for pairs of binding free energies of fitting ES and ASAP-0000214 datasets of MERS-CoV MPro

2D joint marginal probability densities were estimated based on 10,000 MCMC samples generated from the Bayesian posterior for all datasets.

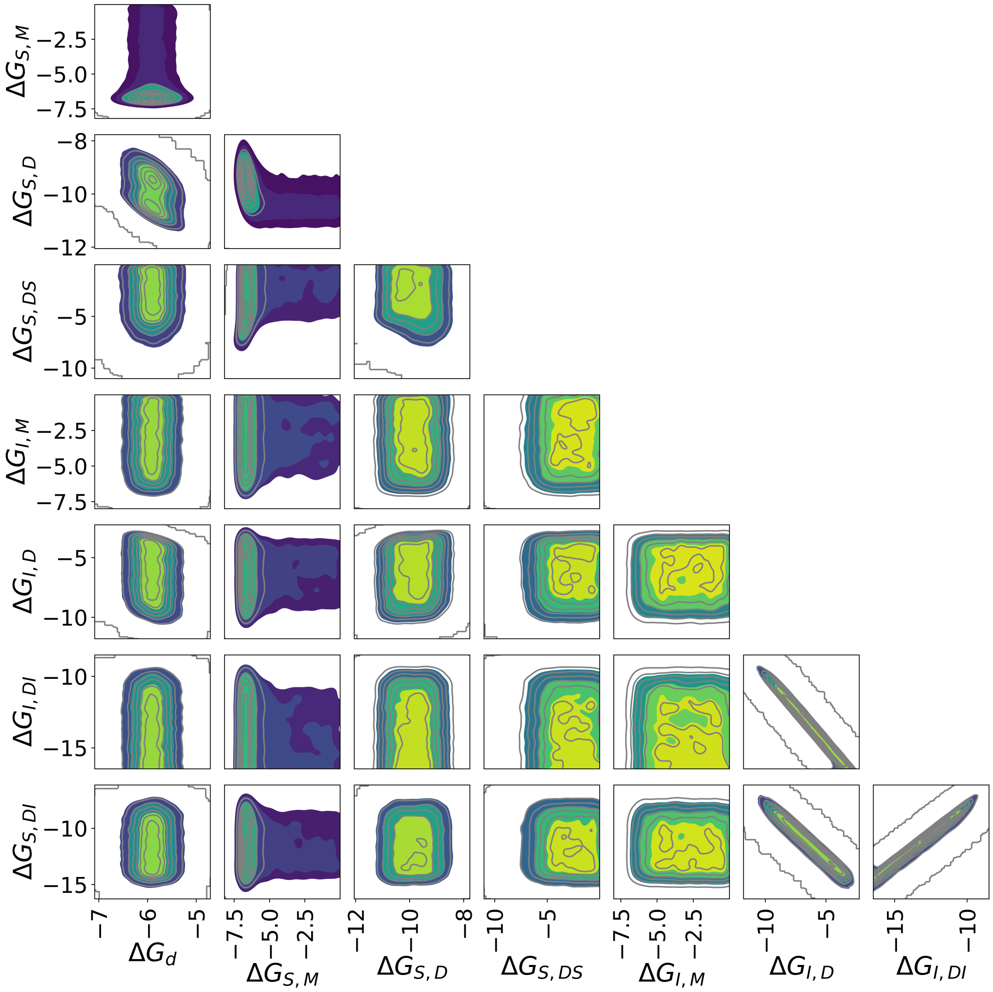

Figure S8. Heat map of the correlation matrix estimated from the Bayesian posterior for binding free energies of fitting ES and ASAP-0000214 datasets of MERS-CoV MPro

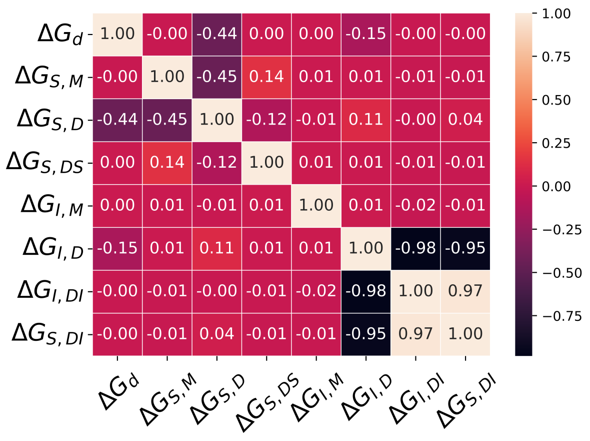

Figure S9. Linear correlation between log$K_{I,D}$, log$K_{S,DI}$, and log$K_{I,DI}$ of fitting ES and ASAP-0000214 datasets of MERS-CoV MPro.

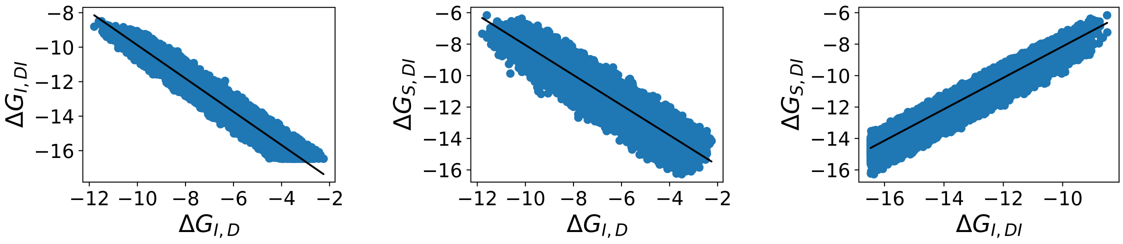

(a) (b) (c)

Figure S10. 1D marginal distributions for rate constants of fitting ES and ASAP-0000214 datasets of MERS-CoV MPro

1D marginal probability densities for kinetic parameters were estimated based on 10,000 MCMC samples generated from the Bayesian posterior for all datasets. Red lines represent 95% HDIs. The green triangle marks the median of the posterior.

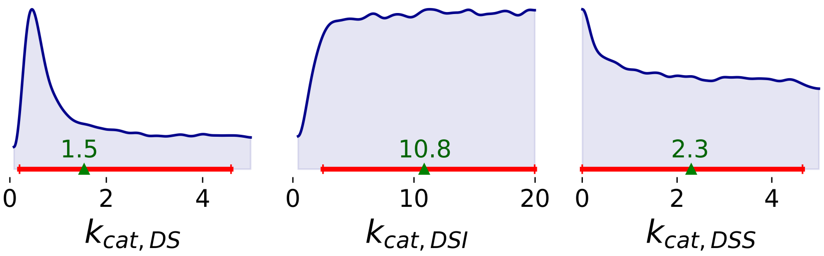

Figure S11. 2D marginal distributions for pairs of rate constants of fitting ES and ASAP-0000214 datasets of MERS-CoV MPro

2D joint marginal probability densities were estimated based on 10,000 MCMC samples generated from the Bayesian posteriors.

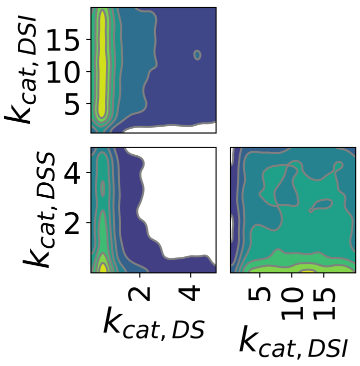

Figure S12. Representative 1D marginal distributions for enzyme concentrations of fitting ES and ASAP-0000214 datasets of MERS-CoV MPro

1D marginal probability densities were estimated based on 10,000 MCMC samples generated from the Bayesian posterior for all datasets. Red lines represent 95% HDIs. The green triangle marks the median of the posterior.

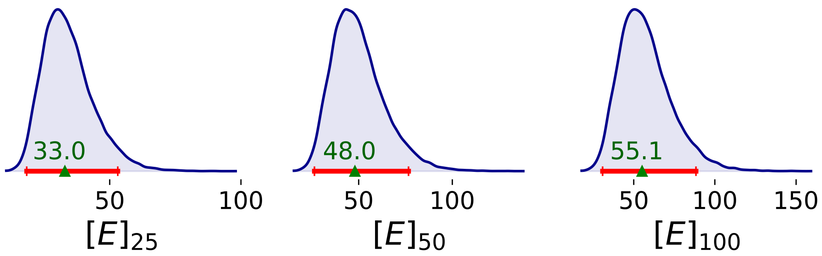

Fitting of ES and all ESI4c datasets

Figure S13. 2D joint marginal distributions for pairs of some binding free energies of fitting ES and all ESI4c datasets

2D marginal probability densitie were estimated based on 1,000 MCMC samples generated from the Bayesian posterior for fitting ES and all ESI4c datasets.

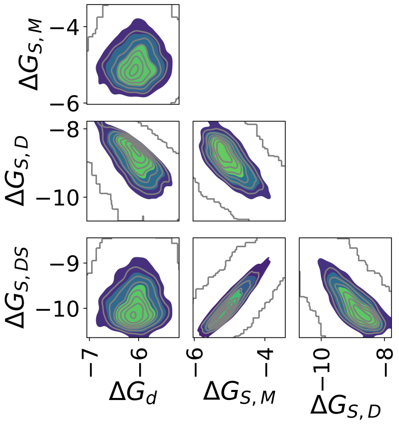

Figure S14. Heat map of the correlation matrix estimated from the Bayesian posterior for some binding free energies of fitting ES and all ESI4c datasets

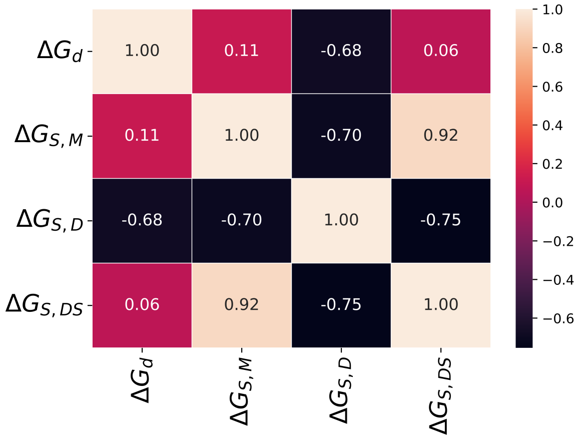

Figure S15. Fit of the model to ES and all ESI4c datasets

Curves in the same colors represent for the datasets in the same plates. Dots are the observed response. X axes are concentrations (M). The theoretical response $y_n * (\boldsymbol{\theta}^{MAP})$ is represented by the dashed line, where $\boldsymbol{\theta}^{MAP}$ is the MAP estimate, the mean of the posterior prediction is solid line, and the 95% posterior predictive interval is the shaded region. For the first plot: 100 nM (brown), 50 nM (red), and 25 nM (black) of the enzyme. For other plots: enzyme 100 nM, substrate 1350 nM (blue); enzyme 100 nM, substrate 750 nM (orange); enzyme 50 nM, substrate 150 nM (purple); enzyme 100 nM, substrate 50 nM (green).

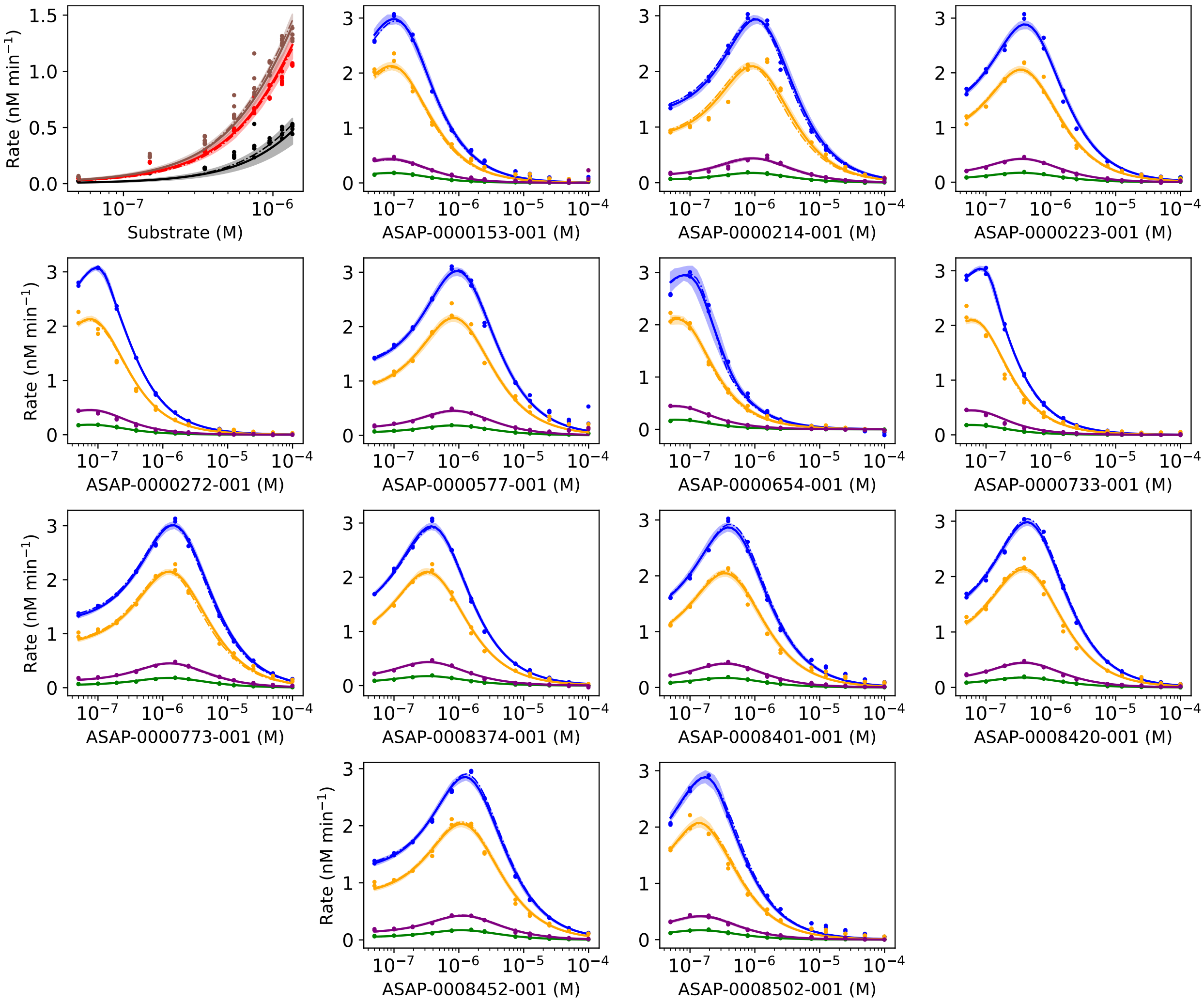

Fitting of ES, all ESI4c, and 3 ESI1c datasets

Figure S16. Convergence of percentiles of the Bayesian posterior for shared parameters for fitting ES, all ESI4c, and 3 ESI1c datasets

1,000 samples were drawn from the Bayesian posterior using the NUTS sampler. Lines correspond to the 5-th (blue circle), 25-th (green square), 50-th (red diamond), 75-th (cyan upward triangle) and 95-th (magenta downward triangle) percentile. The error bars, which are too small to be visible, are standard deviations estimated by 100 bootstrapping samples.

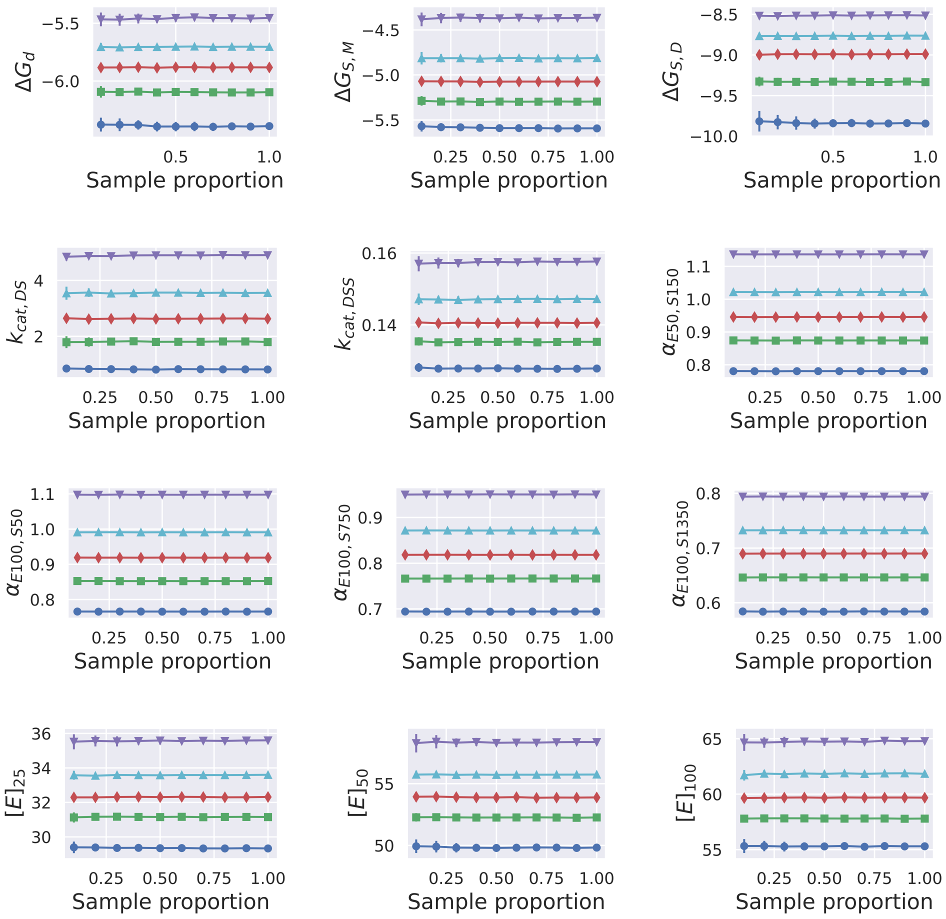

Figure S17. 2D joint marginal distributions for pairs of some binding free energies of fitting ES, all ESI4c, and 3 ESI1c datasets

2D joint marginal probability densities were estimated based on 1,000 MCMC samples generated from the Bayesian posterior for all datasets.

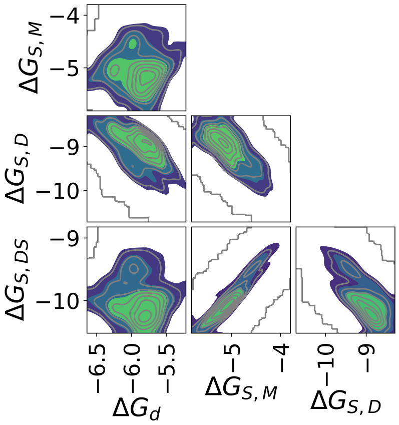

Figure S18. Heat map of the correlation matrix estimated from the Bayesian posterior for some binding free energies for fitting ES, all ESI4c, and 3 ESI1c datasets

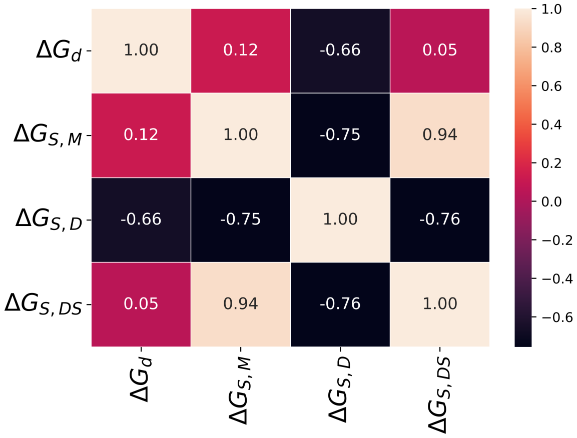

Figure S19. Ratios of rate constants of MERS-CoV MPro

Prior distributions are shown in black line, posterior of fitting datasets of ES + one ESI4c as green dashdot line, ES + all ESI4c as orange dashed line, ES + all ESI4c + three ESI1c as blue solid line. Red lines represent 95% HDI. The red triangle marks the median of the posterior.

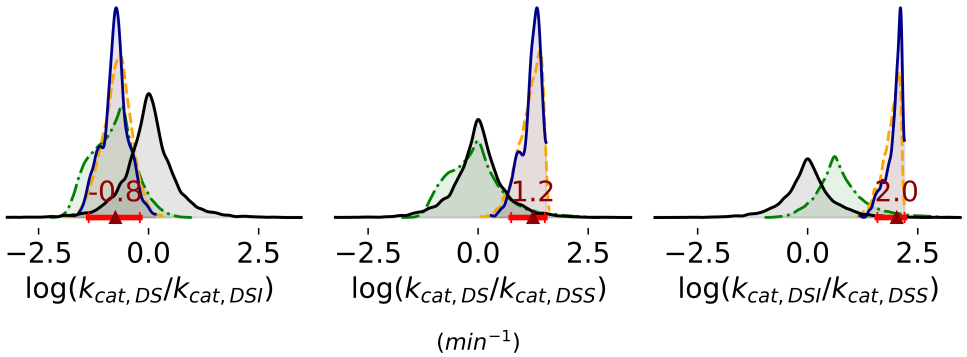

Fitting 1 ESI1c dataset

Figure S20. Convergence of percentiles of the Bayesian posterior for key parameters for fitting one ESI1c dataset

1,000 samples were drawn from the Bayesian posterior using the NUTS sampler. Lines correspond to the 5-th (blue circle), 25-th (green square), 50-th (red diamond), 75-th (cyan upward triangle) and 95-th (magenta downward triangle) percentile. The error bars, which are too small to be visible, are standard deviations estimated by 100 bootstrapping samples.

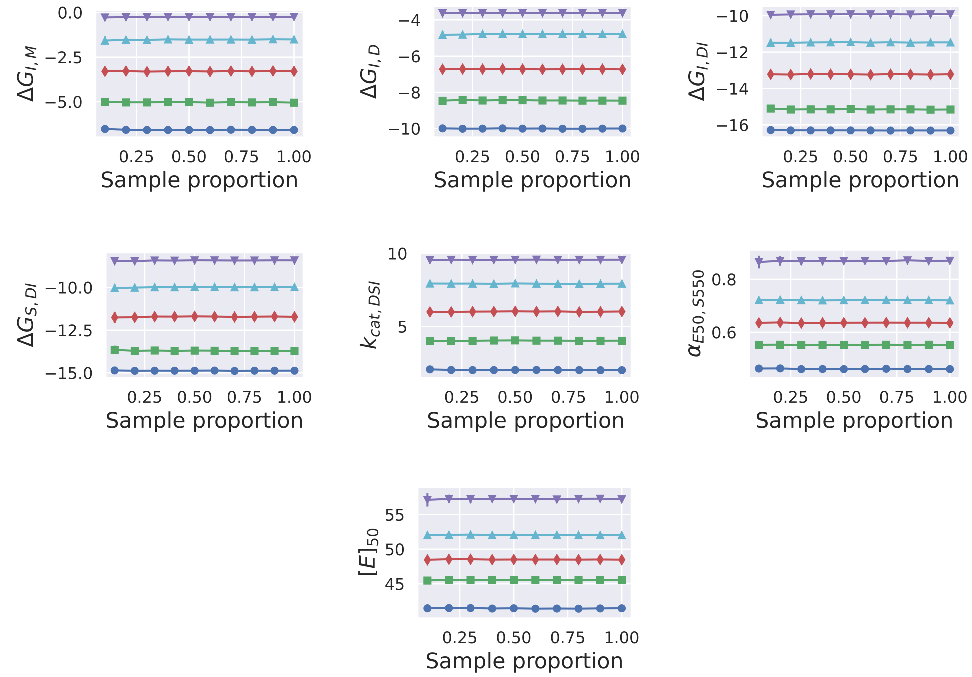

Figure S21. Fit of the model to ESI1c datasets

Dots are the observed response. X axes are concentrations (M). The theoretical response $y_n * (\boldsymbol{\theta}^{MAP})$ is represented by the dashed line, where $\boldsymbol{\theta}^{MAP}$ is the MAP estimate, the mean of the posterior prediction is solid line, and the 95% posterior predictive interval is the shaded region.

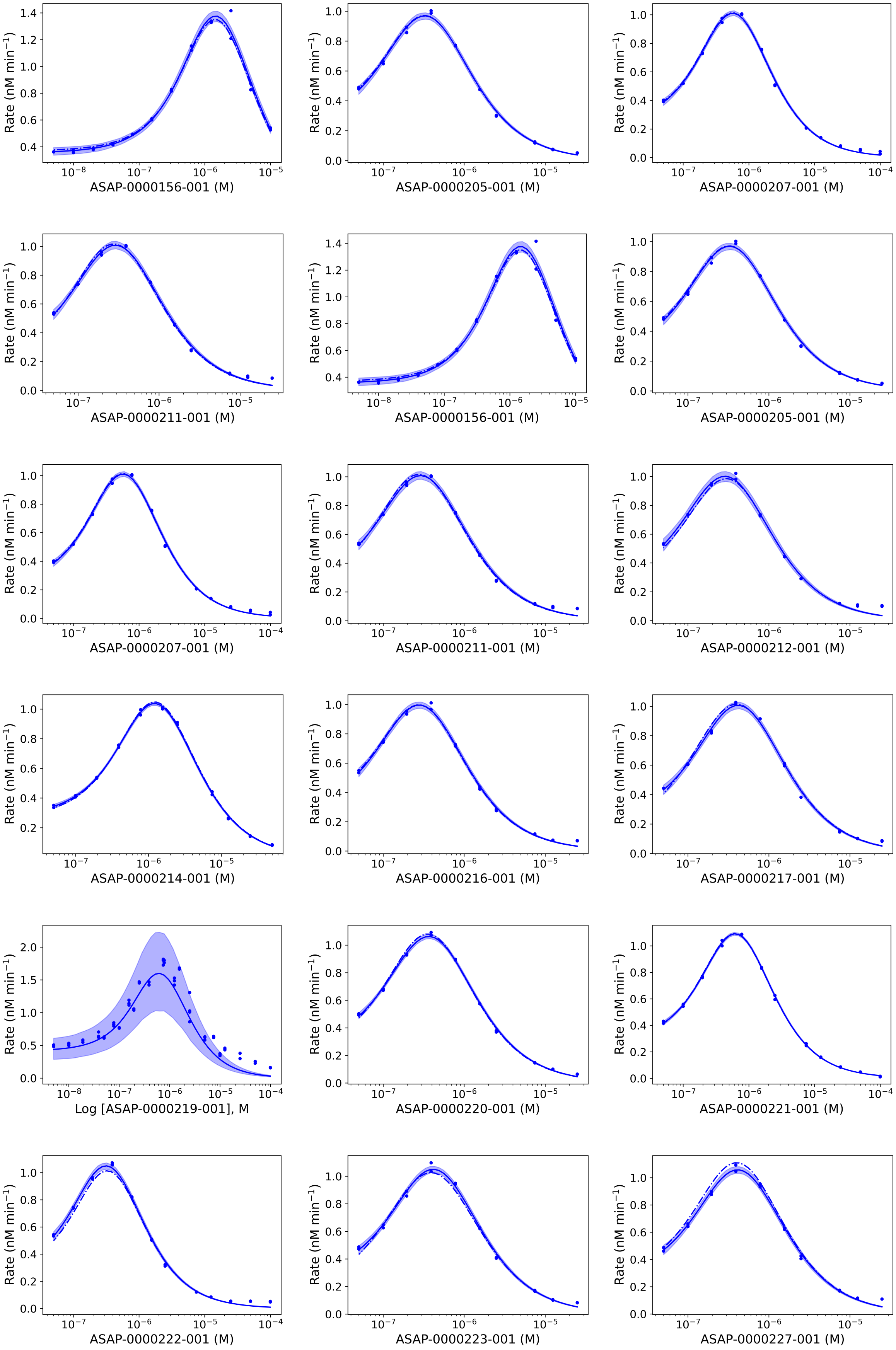
Rate (nM min−1)
ASAP-0000156-001 (M)
ASAP-0000205-001 (M)
ASAP-0000207-001 (M)
ASAP-0000211-001 (M)
ASAP-0000156-001 (M)
ASAP-0000205-001 (M)
ASAP-0000207-001 (M)
ASAP-0000211-001 (M)
ASAP-0000212-001 (M)
ASAP-0000214-001 (M)
ASAP-0000216-001 (M)
ASAP-0000217-001 (M)
Log [ASAP-0000219-001], M
ASAP-0000220-001 (M)
ASAP-0000221-001 (M)
ASAP-0000222-001 (M)
ASAP-0000223-001 (M)
ASAP-0000227-001 (M)

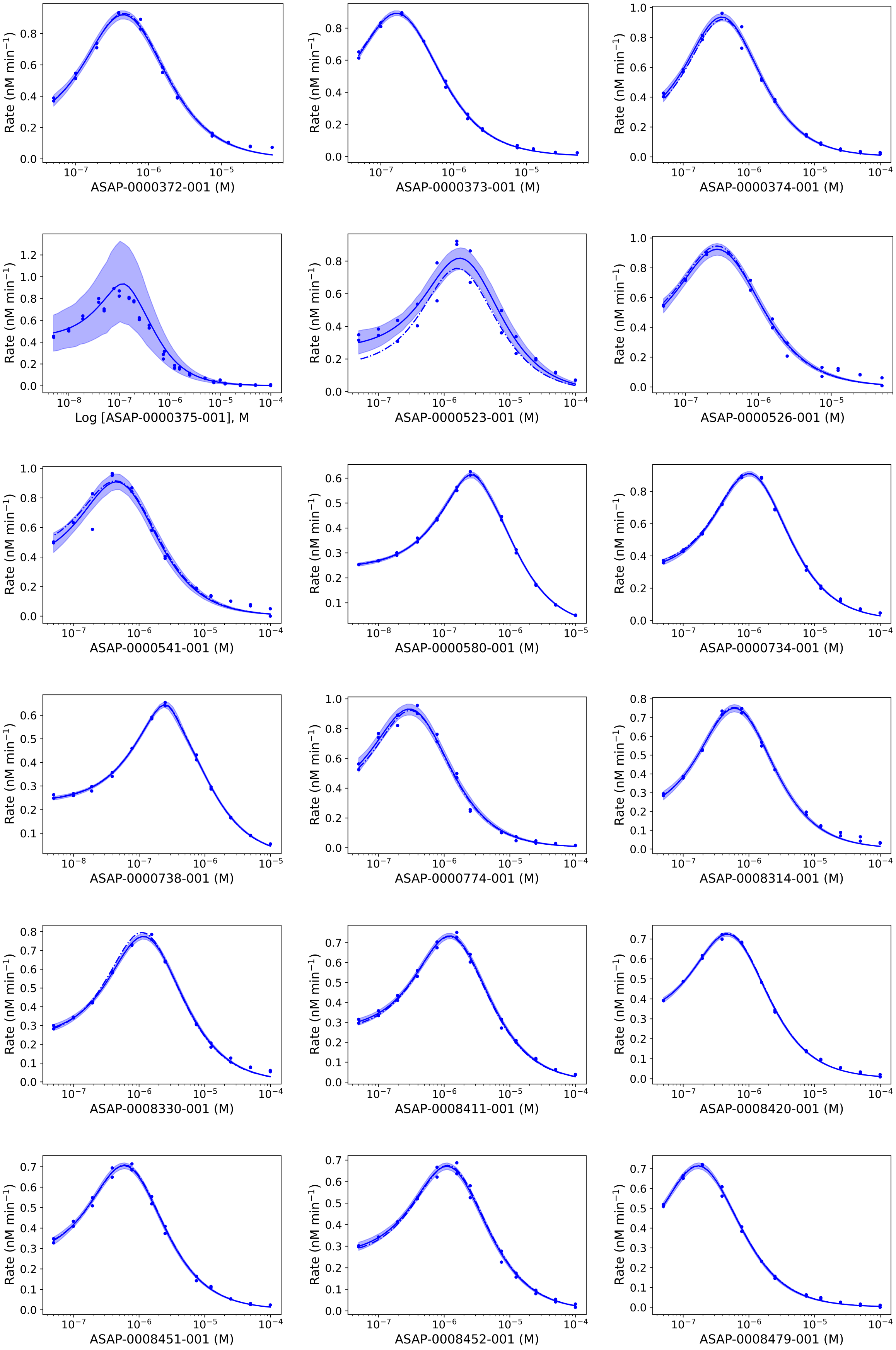
Rate (nM min^{-1})
ASAP-0000372-001 (M)
ASAP-0000373-001 (M)
ASAP-0000374-001 (M)
Log [ASAP-0000375-001], M
ASAP-0000523-001 (M)
ASAP-0000526-001 (M)
ASAP-0000541-001 (M)
ASAP-0000580-001 (M)
ASAP-0000734-001 (M)
ASAP-0000738-001 (M)
ASAP-0000774-001 (M)
ASAP-0008314-001 (M)
ASAP-0008330-001 (M)
ASAP-0008411-001 (M)
ASAP-0008420-001 (M)
ASAP-0008451-001 (M)
ASAP-0008452-001 (M)
ASAP-0008479-001 (M)

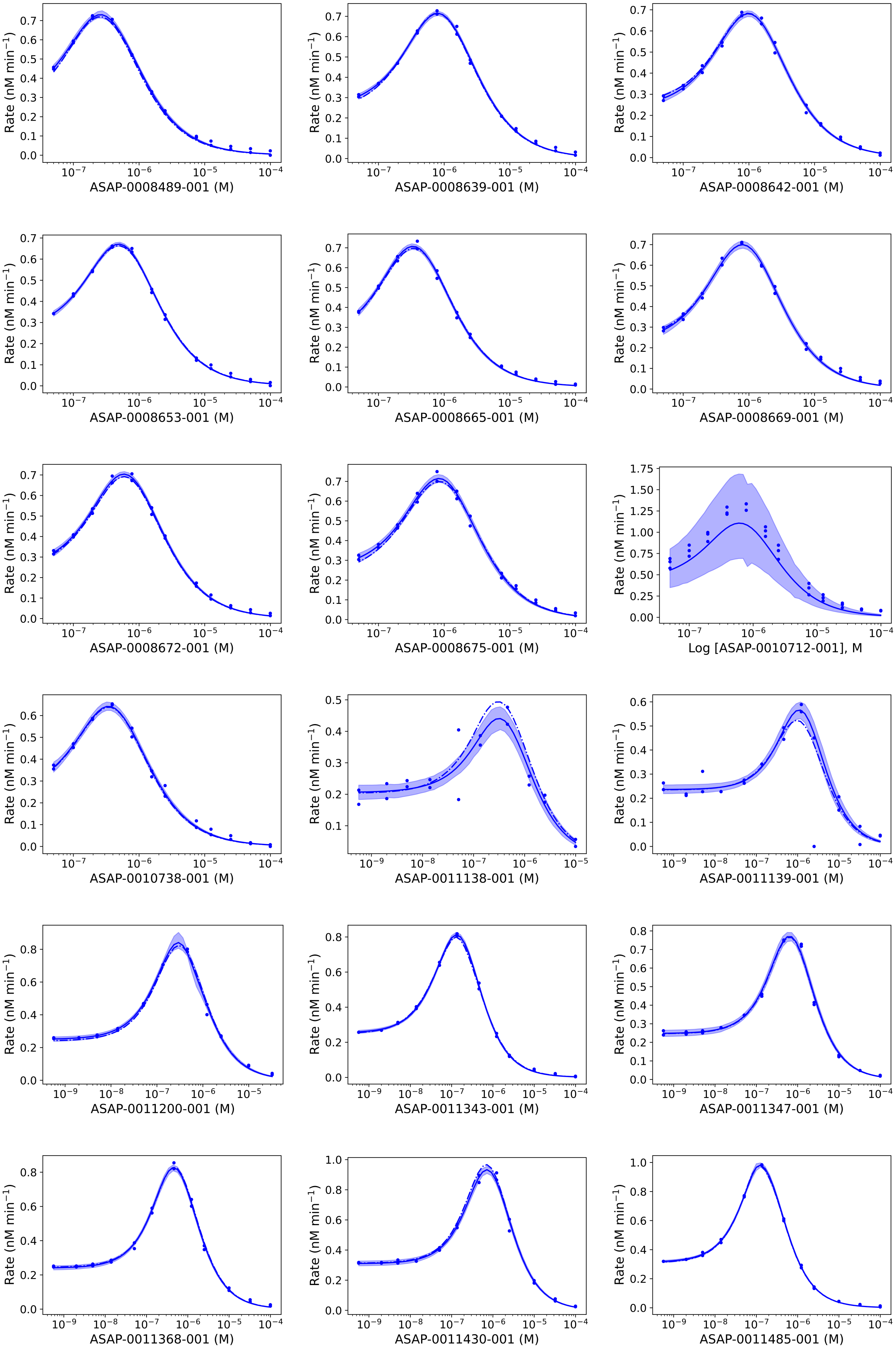
Rate (nM min^{-1})
ASAP-0008489-001 (M)
ASAP-0008639-001 (M)
ASAP-0008642-001 (M)
ASAP-0008653-001 (M)
ASAP-0008665-001 (M)
ASAP-0008669-001 (M)
ASAP-0008672-001 (M)
ASAP-0008675-001 (M)
Log [ASAP-0010712-001], M
ASAP-0010738-001 (M)
ASAP-0011138-001 (M)
ASAP-0011139-001 (M)
ASAP-0011200-001 (M)
ASAP-0011343-001 (M)
ASAP-0011347-001 (M)
ASAP-0011368-001 (M)
ASAP-0011430-001 (M)
ASAP-0011485-001 (M)

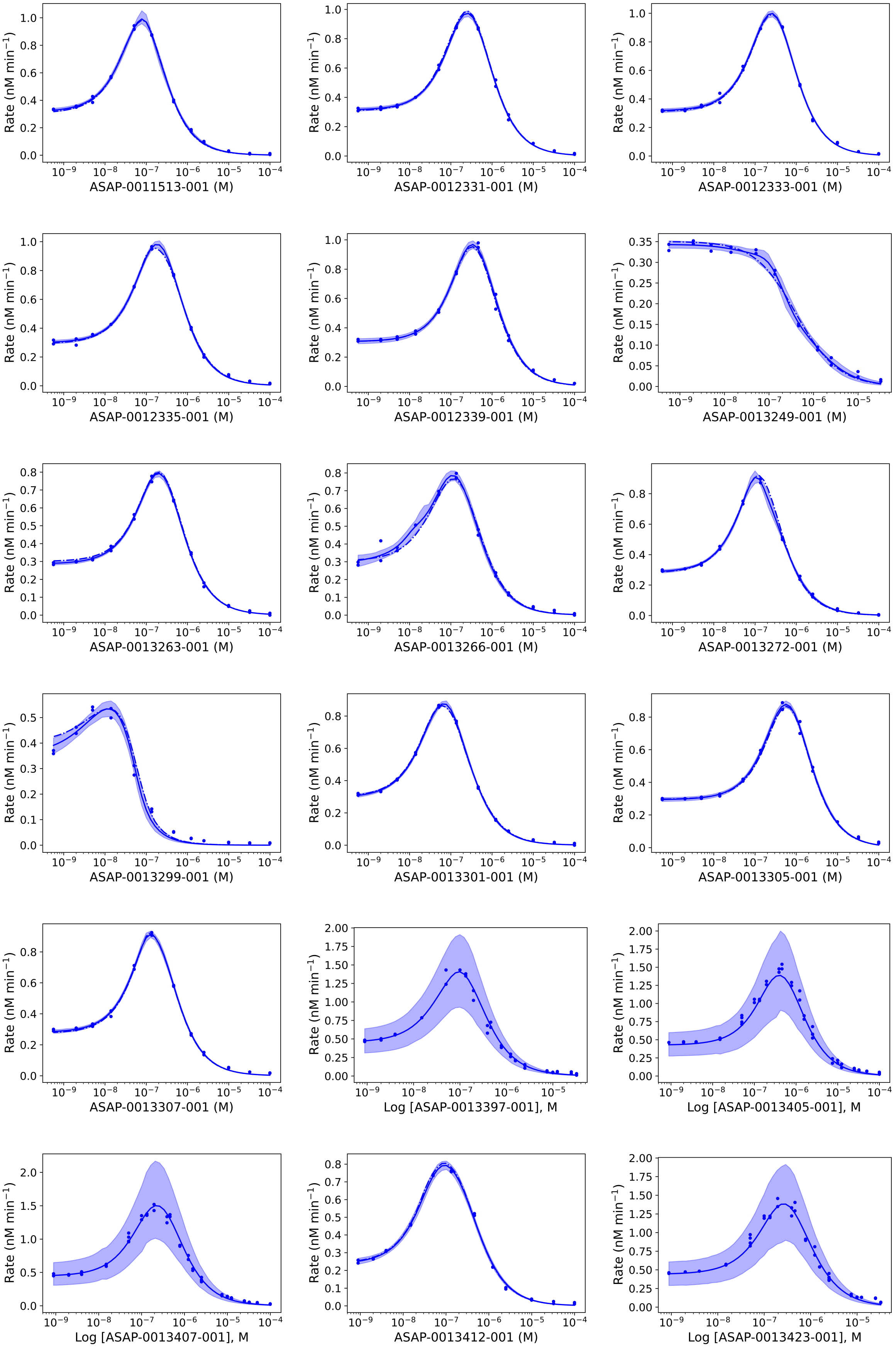
Rate (nM min−1)
ASAP-0011513-001 (M)
ASAP-0012331-001 (M)
ASAP-0012333-001 (M)
ASAP-0012335-001 (M)
ASAP-0012339-001 (M)
ASAP-0013249-001 (M)
ASAP-0013263-001 (M)
ASAP-0013266-001 (M)
ASAP-0013272-001 (M)
ASAP-0013299-001 (M)
ASAP-0013301-001 (M)
ASAP-0013305-001 (M)
ASAP-0013307-001 (M)
Log [ASAP-0013397-001], M
Log [ASAP-0013405-001], M
Log [ASAP-0013407-001], M
ASAP-0013412-001 (M)
Log [ASAP-0013423-001], M

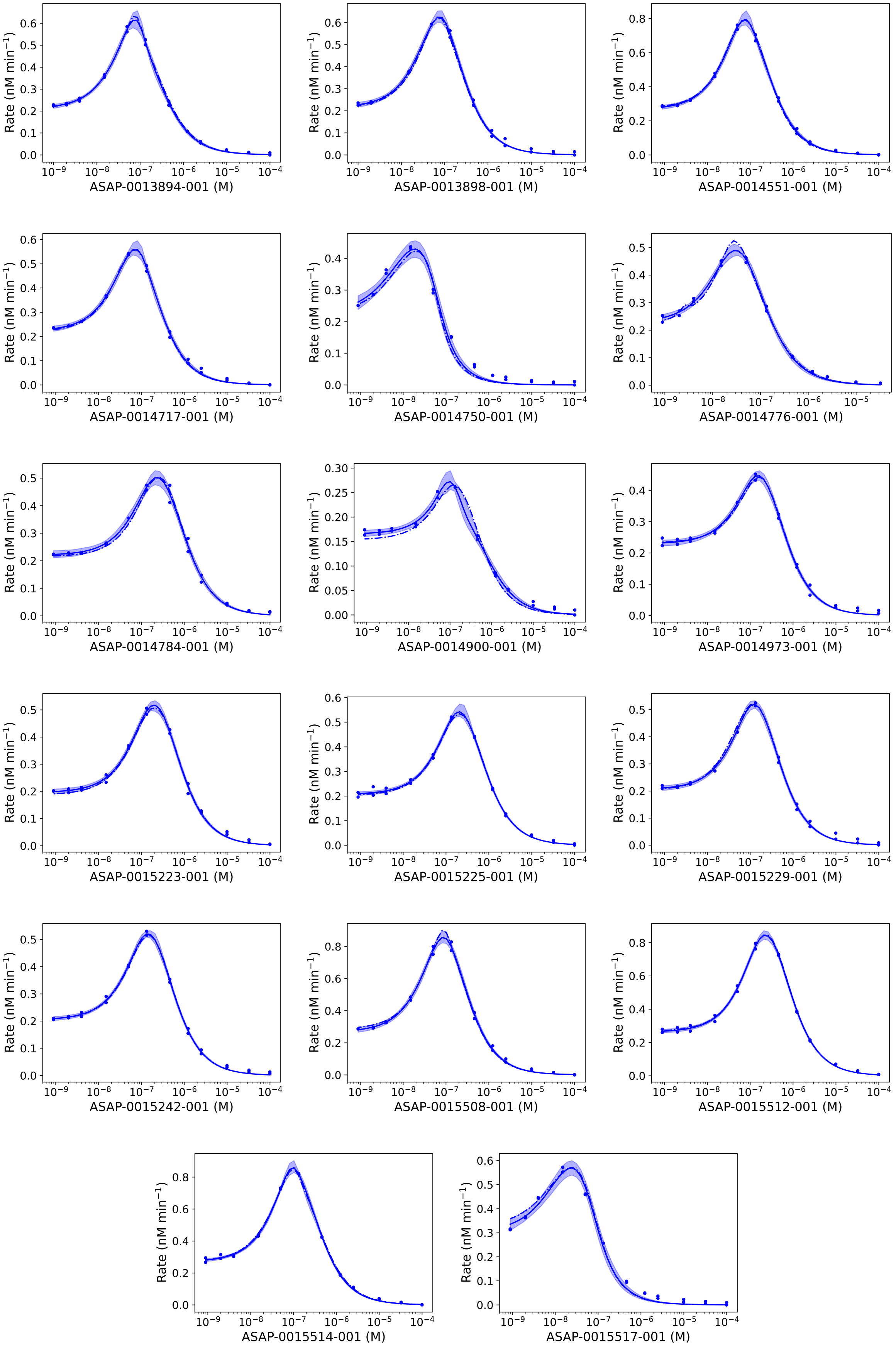
Rate (nM min^{-1})
ASAP-0013894-001 (M)
ASAP-0013898-001 (M)
ASAP-0014551-001 (M)
ASAP-0014717-001 (M)
ASAP-0014750-001 (M)
ASAP-0014776-001 (M)
ASAP-0014784-001 (M)
ASAP-0014900-001 (M)
ASAP-0014973-001 (M)
ASAP-0015223-001 (M)
ASAP-0015225-001 (M)
ASAP-0015229-001 (M)
ASAP-0015242-001 (M)
ASAP-0015508-001 (M)
ASAP-0015512-001 (M)
ASAP-0015514-001 (M)
ASAP-0015517-001 (M)

Correlation Analysis

Figure S22. Correlogram of inhibition, control, dimer pIC90 and cellular pEC90

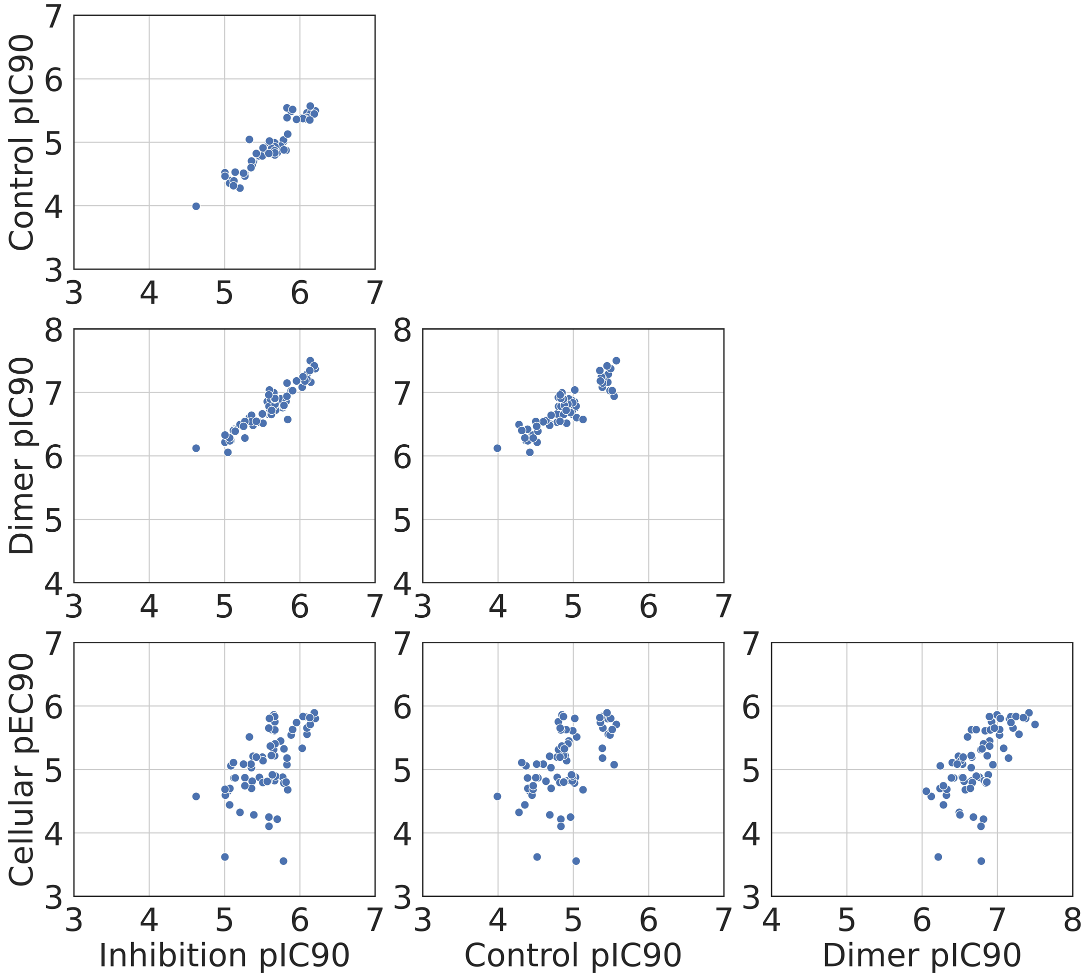

Table S1. Correlation matrix of biochemical pIC90 and cellular pEC90 by Pearson R, Spearman ρ, and Kendall τ

Pearson R	Inhibition pIC90	Control pIC90	Dimer pIC90
Control $pIC90$	0.954 ± 1.500E-2		
Dimer $pIC90$	0.873 ± 7.405E-2	0.858 ± 6.712E-2	
Cellular $pEC90$	0.680 ± 7.374E-2	0.670 ± 7.343E-2	0.698 ± 6.266E-2

Spearman ρ	Inhibition pIC90	Control pIC90	Dimer pIC90
Control $pIC90$	0.910 ± 2.578E-2		
Dimer $pIC90$	0.896 ± 5.474E-2	0.861 ± 5.055E-2	
Cellular $pEC90$	0.685 ± 6.430E-2	0.639 ± 7.507E-2	0.748 ± 4.883E-2

Kendall τ	Inhibition pIC90	Control pIC90	Dimer pIC90
Control $pIC90$	0.754 ± 4.001E-2		
Dimer $pIC90$	0.776 ± 5.488E-2	0.698 ± 5.590E-2	
Cellular $pEC90$	0.505 ± 5.642E-2	0.466 ± 6.426E-2	0.554 ± 4.683E-2

Table S2. Correlation matrix of biochemical pIC90 and cellular pEC90 by RMSD and aRMSD

RMSD	Inhibition pIC90	Control pIC90	Dimer pIC90
Control $pIC90$	0.684 ± 1.565E-2		
Dimer $pIC90$	0.613 ± 5.828E-2	0.296 ± 8.985E-2	
Cellular $pEC90$	0.623 ± 6.124E-2	0.477 ± 3.895E-2	0.447 ± 5.074E-2

aRMSD	Inhibition pIC90	Control pIC90	Dimer pIC90
Control $pIC90$	0.132 ± 1.499E-2		
Dimer $pIC90$	0.250 ± 1.011E-1	0.264 ± 8.720E-2	
Cellular $pEC90$	0.416 ± 5.399E-2	0.425 ± 5.247E-2	0.429 ± 5.921E-2

Table S3. p-value in comparison of correlation for $pIC50$s

Pearson R	Cellular vs. Inhibition	Cellular vs. Control
Cellular vs. Control	0.191	
Cellular vs. Dimer	0.181	0.242

Spearman rho	Cellular vs. Inhibition	Cellular vs. Control
Cellular vs. Control	0.121	
Cellular vs. Dimer	0.018	0.004

Kendall τ	Cellular vs. Inhibition	Cellular vs. Control
Cellular vs. Control	0.171	
Cellular vs. Dimer	0.039	0.0191

RMSD	Cellular vs. Inhibition	Cellular vs. Control
Cellular vs. Control	8.919E-22	
Cellular vs. Dimer	1.694E-10	0.250

aRMSD	Cellular vs. Inhibition	Cellular vs. Control
Cellular vs. Control	0.037	
Cellular vs. Dimer	2.174E-04	0.002

Table S4. p-value in comparison of correlation for $pIC90$s

Pearson R	Cellular vs. Inhibition	Cellular vs. Control
Cellular vs. Control	0.184	
Cellular vs. Dimer	0.128	0.077

Spearman *rho*	Cellular vs. Inhibition	Cellular vs. Control
Cellular vs. Control	0.026	
Cellular vs. Dimer	0.002	6.868E-06

Kendall τ	Cellular vs. Inhibition	Cellular vs. Control
Cellular vs. Control	0.027	
Cellular vs. Dimer	0.005	3.523E-05

RMSD	Cellular vs. Inhibition	Cellular vs. Control
Cellular vs. Control	1.959E-11	
Cellular vs. Dimer	1.204E-12	0.024

aRMSD	Cellular vs. Inhibition	Cellular vs. Control
Cellular vs. Control	0.168	
Cellular vs. Dimer	0.142	0.215

Data

`SI.xlsx`

includes enzyme velocities for the datasets ES, ESI4c, and ESI1c.
